# Distribution and Clinical Analysis of EpCAM+/Vimentin+ Circulating Tumor Cells in High-Risk Population and Cancer Patients

**DOI:** 10.3389/fonc.2021.642971

**Published:** 2021-06-08

**Authors:** Chunjin Huang, Sheng Ding, Chunyan Huang, Feng Pan, Xiaodong Liu, Haijiao Zhang, Jian Zhou, Xiaofei Liang, Xinyan Wang, Ping Song

**Affiliations:** ^1^ Department of General Surgery, Huadong Hospital Affiliated to Fudan University, Shanghai, China; ^2^ Department of Stomatology, Xinhua Hospital Affiliated to Shanghai Jiaotong University School of Medicine, Shanghai, China; ^3^ Department of anesthesia, Zhabei Central Hospital of Shanghai, Shanghai, China; ^4^ Department of Orthopedics, Zhabei Central Hospital of Shanghai, Shanghai, China; ^5^ Project Department, Huzhou Lieyuan Medical Laboratory Company Ltd, Huzhou, China

**Keywords:** cancer, circulating tumor cells, epithelial cell adhesion molecular, vimentin, high-risk population

## Abstract

Circulating Tumor Cells (CTCs) are already present in the peripheral blood of patients with early tumors and even precancerous lesions. The objective of this study was to determine the count of CTCs in peripheral blood from high-risk population(HRP), healthy subjects and patients with Pan-cancer. The CTCs in the peripheral blood from HRP and cancer patients were enriched and identified based on the positive sorting method by epithelial cell adhesion molecular (EpCAM) liposome magnetic bead (Ep-LMB) and Vimentin liposome magnetic bead (Vi-LMB). Simultaneously, further analysis was carried out focusing on the clinical characteristics of patients by collecting the peripheral blood samples from healthy subjects as the parallel control. According to the results, the prepared LMBs had high specificity and stability, resulting in an average (Av) proliferation rate of over 90% for each cell line, and the average capture rate of higher than 80%. In terms of CTCs count detection in clinical blood samples, the average count was 0.9 (Ep: Av=0.6, Vi: Av=0.3), 2.4 (Ep: Av=1.4, Vi: Av=0.8) and 7.3 (Ep: Av=4.0, Vi: Av=3.3) in healthy subjects, HRP and total cancer patients, respectively. Besides, there was no obvious difference in the average count of CTCs among patients with different cancer types. While count of CTCs in the aforementioned cancer patients was statistically different from that in healthy subjects and patients with HRP. The survival time of cancer patients whose number of CTCs is greater than the average is significantly increased. Collectively, the study confirmed that CTCs can achieve early tumor detection and auxiliary diagnosis, and its number is related to the occurrence and development of tumors, and CTCs can be detected in HRP and sub-health population.

## Introduction

Circulating tumor cells (CTCs) are cells originating from the primary lesion that abscission to the peripheral blood; they have a putative role in metastasis formation (1, 2). Their presence in patients with solid tumors at all disease stages is well established (3, 4), and they have the same cytomorphologic features as the solid tumor (5). Detection of CTCs in the peripheral blood of cancer patients can be prognostic and may have a large impact on the development of new strategies for cancer treatment (6, 7). CTCs have recently emerged as important potential biomarkers of diagnosis, evaluation of treatment effect, and prognosis in cancers including lung cancer (8),gastric cancer (9), colorectal cancer (10), liver cancer (11) and esophageal cancer (12).

Studies have found that the formation of CTCs was related to epithelial mesenchymal transition(EMT). EMT is the process of conversion from an epithelial to a mesenchymal phenotype, resulting in expression of mesenchymal markers (Vimentin) and loss of epithelial markers (epithelial cell adhesion molecular, EpCAM). The recent methods of epithelial cell adhesion molecule (EpCAM)-based CTCs analysis showed limitations to detect CTCs in patients with tumors (13, 14), because some CTCs express only epithelial or mesenchyme markers (15, 16). The vimentin are highly expressed in a variety of tumor cells, especially in tumor cells with EMT (17). Therefore, vimentin may be a potential target for capturing CTCs in patients with tumor. A novel EpCAM+/Vimentin+ -based CTCs analysis method had been developed on the basis of this fact.

An accurate counting of CTCs can be used in the clinical application of auxiliary means for tumor staging (18), curative effect monitoring (19), recurrence prediction (20), prognosis evaluation (21, 22), etc. For instance, a previous study found that >5 CTCs were detected in 7.5 mL blood samples, which generally indicated poor clinical results, i.e., significantly shortened overall survival and progression free survival. It suggested that the count of CTCs can reflect the therapeutic effect and the survival of cancer patients (23, 24). Meanwhile, Algizawy, et al. carried out a study by enrolling 85 breast cancer patients and confirmed that the count of CTCs can be used as an auxiliary means to monitor the progress of the disease (25).

With respect to the above, the present study was conducted focusing on the capture of CTCs in peripheral blood of healthy subjects, patients with high-risk population (HRP) and those with different types of cancer on the basis of the preparation of EpCAM+Vimentin specific immunomagnetic beads, with the aim to study the difference of CTCs counts of non-cancer patients with healthy subjects and cancer patients.

## Materials and Methods

### Objects of Study

The objects of study were 32 cases of HRP diagnosed in our hospital from 2017 to 2019, HRP is for patients with disease(Including: lumbar disc herniation, coronary heart disease, diabetes, chronic gastritis, appendicitis, acute bronchitis, rheumatoid arthritis etc.), as well as 37 cases of lung cancer, 33 cases of gastric cancer, 38 cases of colorectal cancer, 32 cases of liver cancer, 34 cases of esophageal cancer and 25 cases of healthy volunteers from Huzhou Lieyuan Medical Laboratory Co., Ltd. The method of collecting blood samples is to collect 3.75 mL of peripheral blood of patients with medical anticoagulant blood vessels, and the anticoagulant is EDTA K2. This study was approved by the Ethics Committee of Zhabei Central Hospital of Shanghai (ZBLL20180823). Participants were gave written consent after receiving verbal and written information.

### Cell Lines

MKN-45 gastric cancer cells, Li-7 liver cancer cells, A549 lung cancer cells, KYSE450 esophageal cancer cells, and SW116 colorectal cancer cells were purchased from Shanghai Cell Bank of Chinese Academy of Sciences. Cells were cultured in RPMll640 medium containing 10% fetal bovine serum in a 5% CO_2_ constant-temperature incubator at 37°C.

### Materials and Instruments

DMEM culture medium, fetal bovine serum and trypsin (Gibco); Fe_3_O_4_ solution, hexadecyl-quaternized (carboxymethyl) chitosans (HQCMC), CK-FITC, CD45-PE and DAPI (Huzhou Lieyuan Medical Laboratory Co., Ltd.); 1,2 dioleolyl-sn-glycero-3-phosphatidylcholine (DOPC), glycidyl hexadecyl dimethylammonium chloride (GHDC), cholesterol, N-hydroxysuccinimide (NHS), and 1-ethyl-3-(3-dimethyl aminopropyl)-carbodiimide (EDC) (Sinopharm); EpCAM antibody and Vimentin antibody (CST); BI-90Plus Nanoparticle Size Analyzer/Zeta potentiometer (Brookhaven, USA); OLYMPUS B×61 fluorescence microscope (Olympus, Japan); and LDJ9600-1 magnetic property measurement system (MPMS) (Digital Instruments Inc., USA).

### Preparation of Liposome Immunomagnetic Beads

Three micron-nano-sized liposome immunomagnetic beads in this study were prepared by inverted evaporation. Taking epithelial cell adhesion molecular (EpCAM) liposome magnetic bead (Ep-LMB) as an example, with dichloromethane as a co-solvent, the liposome matrix was prepared using cholesterol, 1,2-dioleoyl-sn-glycero-3-phosphocholine (DOPC), glycidyl hexadecyl dimethylammonium chloride (GHDC) and hexadecyl-quaternized (HQCMC). Fe_3_O_4_ was suspended in 0.1 mol/L PBS solution (pH=7.4), and dropped into the liposome matrix. Using a probe-type ultrasonic instrument, the mixed solution was subject to ultrasonic vibration to realize a complete emulsification, and the liposome magnetic bead (LMB) was obtained. A proper amount of EpCAM antibodies was dissolved in 10mL isopropanol, followed by the addition of coupling N-Hydroxysuccinimide (NHS) and 1-ethyl-3 -(3-dimethyl-aminopropyl) carbodiimide, hydrochloride (EDC). After that, the mixed solution was stirred at 4°C for 24 h at a constant speed to obtain EpCAM antibodies modified LMB, named Ep-LMB. It was added with distilled water and diluted (1mg/mL) for further usage. The preparation of Vimentin liposome magnetic bead (Vi-LMB) followed the same method as that of Ep-LMB.

### Characterization Tests

The prepared Vi-LMB and Ep-LMB were tested by MPMS; BI-90Plus Nanoparticle Size Analyzer/Zeta potentiometer were used for the test of particle size and potential of LMB; the ultraviolet absorption spectrum of LMB was scanned by UV spectrophotometer, and its morphology was observed by atomic force microscope (AFM).

### Cytotoxicity Test

The cultured cancer cells were digested into suspension and counted with Hemocytometer. The cells were added into 96-well plate, with 1,000 cells per well, and the medium was 200μL. In the cell sampling treatment, the addition amount of Vi-LMB and Ep-LMB was 5μg/mL, 10μg/mL, 20μg/mL, 30μg/mL, 40μg/mL, 50μg/mL and 60μg/mL, respectively. A control group was established with DMSO, with 3 repetitions for each concentration of each LMB. MTT test was started following 72 h of culture of the 96-well plate in CO_2_ incubator. After incubation in CO_2_ incubator for 2 h, the cell proliferation rate was calculated by measuring the absorbance of each well at the wavelength of 560nm by using Multiskan Spectrum.

### *In Vitro* Simulation of CTCs Capture Test

The cultured A549 cells were digested into suspension and counted with Hemocytometer, with 100 cells in 3.75mL PBS. In Ep-LMB group, 5, 10, 15, 20 and 25μL 1μg/μL Ep-LMB were added separately, while 5, 10, 15, 20 and 25μL 1μg/μL Vi-LMB were added respectively in Vi-LMB group. Three repetitions were set for magnetic separation and capture of cells in each group. The capture efficiency was calculated by counting using FITC labeled CK19 monoclonal antibody (CK19-FITC), DAPI staining solution and PE labeled CD45 antibody (CD45-PE) staining. The addition amount with the maximum capture rate was obtained, followed by verification in different cancer cells. Further study was performed on the total capture rate of Ep-LMB combined with Vi-LMB in different cells. Simultaneous verification was carried out in terms of the stability of Ep-LMB and Vi-LMB to different cell lines. Finally, through the patient blood simulation system, the above methods were used to add different tumor cells to verify the separation efficiency.

### CTCs Capture and Counting of Clinical Blood Samples

An amount of 3.75mL peripheral blood samples were added to 20μL Vi-LMB for capture, followed by capture with the addition of 20μL Ep-LMB. After capture, magnetic separation was carried out using 10µL FITC labeled CK19 monoclonal antibody (CK19-FITC), 20µL DAPI staining solution and 10µL PE labeled CD45 antibody (CD45-PE) staining in dark for 15 min after well mixing. At the end of staining, magnetic separation was conducted after the LMB was washed with 1mL distilled water. Following magnetic separation, discarding of the solution and repeated washing twice, 20µL solution was mixed with 20µL distilled water in the centrifugal tube, and the mixture was applied uniformly into the polylysine treated anti-off slides. After the droplets dried naturally, the slides were observed and counted under the fluorescence microscope.

### Statistical Analysis

SPSS21.0 statistical software was used to analyze the data (mean ± standard deviation) of this study. One-way analysis of variance was used for comparison at different time points, and SNK test was utilized for pairwise comparison. Besides, comparison between groups applied *t* test. P<0.05 (*P<0.05; **P<0.01; ***P<0.001) meant that the difference was statistically significant.

## Results

### CTCs Capture and Counting Analysis

**Figure 1:**
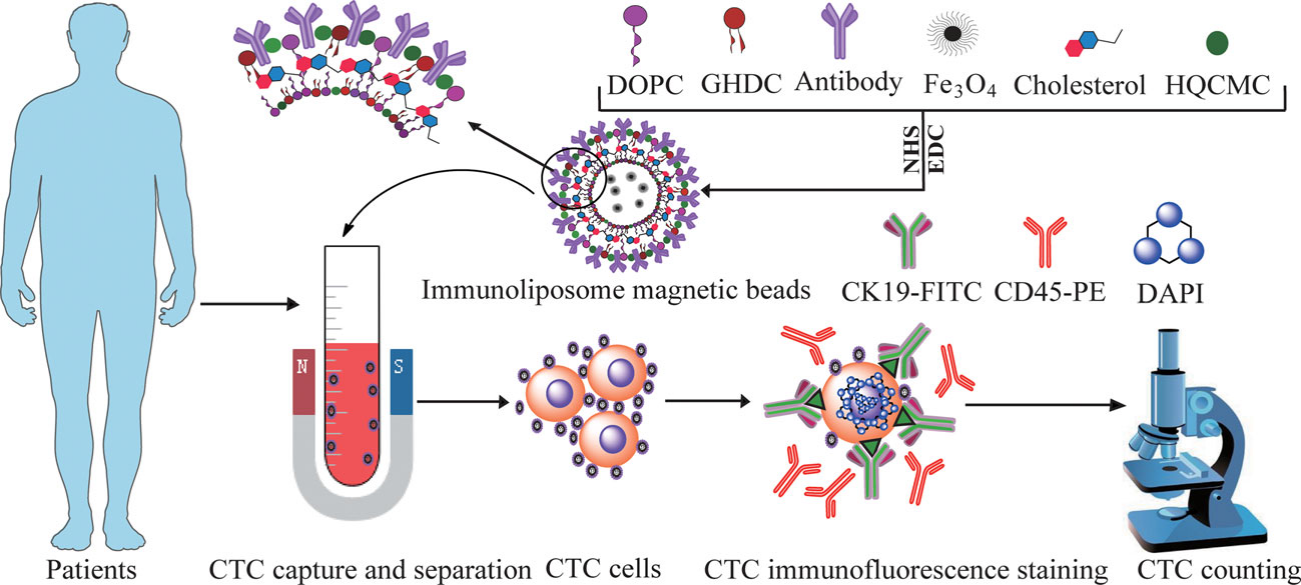
Clinical blood CTCs and detection process.

The CTCs in the 3.75mL peripheral blood from HRP and cancer patients were enriched and identified based on the positive sorting method by Ep-LMB and Vi-LMB. Simultaneously, further analysis was carried out focusing on the clinical characteristics of patients by collecting the 3.75mL peripheral blood samples from 25 healthy subjects as the parallel control. Clinical blood CTCs and detection process in **Figure 1**.

### Characterization of Liposome Immunomagnetic Beads

**Figure 2:**
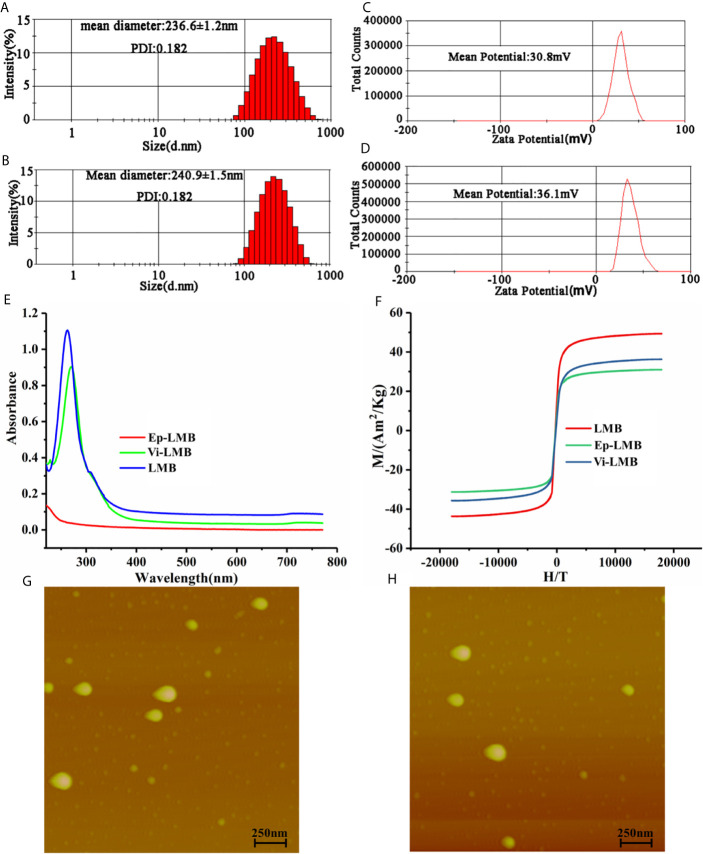
Immunomagnetic bead characterization tests: **(A)** Particle size distribution of Ep-LMB; **(B)** Particle size distribution of Vi-LMB; **(C)** Potential test map of Ep -LMB; **(D)** Potential test map of Vi-LMB; **(E)** Ultraviolet scanning spectrum of Ep-LMB, Vi-LMB and LMB; **(F)** Hysteresis curve; **(G)** Atomic force test results of Ep-LMB; **(H)** Atomic force test results of Vi-LMB.

**Figure 2** shows the characterization results of three magnetic beads prepared. **Figures 2A, B** described the particle size distribution pf Ep-LMB and Vi-LMB, with corresponding average (Av) particle size of 236.6 ± 1.2nm and 240.9 ± 1.5nm, respectively. **Figures 2G, H** are atomic force test results of Ep-LMB and Vi-LMB. The two magnetic microspheres were observed to be spherical with different sizes and regular shapes without agglomeration. Furthermore, **Figures 2C, D** display the potential distribution of Ep-LMB and Vi-LMB. Both magnetic microspheres were positively charged that might benefit the dispersed beads in a hydrophilic solution. The ultraviolet scanning results of immunomagnetic beads are shown in **Figures 2E**. There existed no absorption peak at 280nm for LMB, but absorption peaks at 280nm for both Vi-LMB and Ep-LMB, which had the characteristics of protein ultraviolet absorption. It suggested that EpCAM and vimentin antibodies were indeed grafted to the surface of the magnetosphere. **Figure 2F** is the hysteresis curve of Vi-LMB and Ep-LMB. The magnetic properties of liposomes coated with antibody were much higher than that of liposome immunomagnetic beads with antibody grafted. Further comparative analysis indicated that Fe_3_O_4_ was coated by liposome and then coupled with antibody. Its intensity of magnetism was weakened to some extent in the later stage, suggesting that Fe_3_O_4_ was successfully coated by liposome and grafted with antibody.

### Cell Cytotoxicity

**Figure 3:**
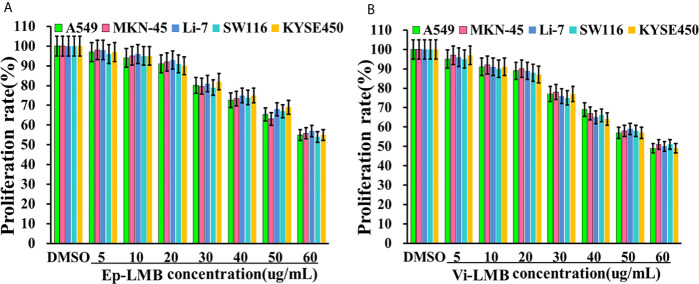
The effect of immunomagnetic beads on the activity of A549 cells: **(A)** Effect of Ep-LMB on the proliferation rate of different cancer cells; **(B)** Effect of Vi-LMB on the proliferation rate of different cancer cells.

**Figures 3A, B** show the results of cell proliferation rate of Ep-LMB and Vi-LMB treated with different concentrations. When the concentration of Ep-LMB and Vi-LMB was below 20μg/mL, their toxicity to cancer cells was relatively small, with the cell proliferation rate of each cell line of >90%. The cell proliferation rate decreased with the increase of the concentration of magnetic beads, indicating that excessive concentration of magnetic beads might have certain toxicity to cells. Furthermore, when the concentration of Ep-LMB and Vi-LMB was 60μg/mL, the proliferation rate of each cell line was still more than 60% and 50%, respectively. It revealed that both Ep-LMB and Vi-LMB might be less toxic despite the presence of cytotoxicity to each cell line.

### Capture Efficiency of Vi-LMB and Ep-LMB for Cancer Cells

**Figure 4:**
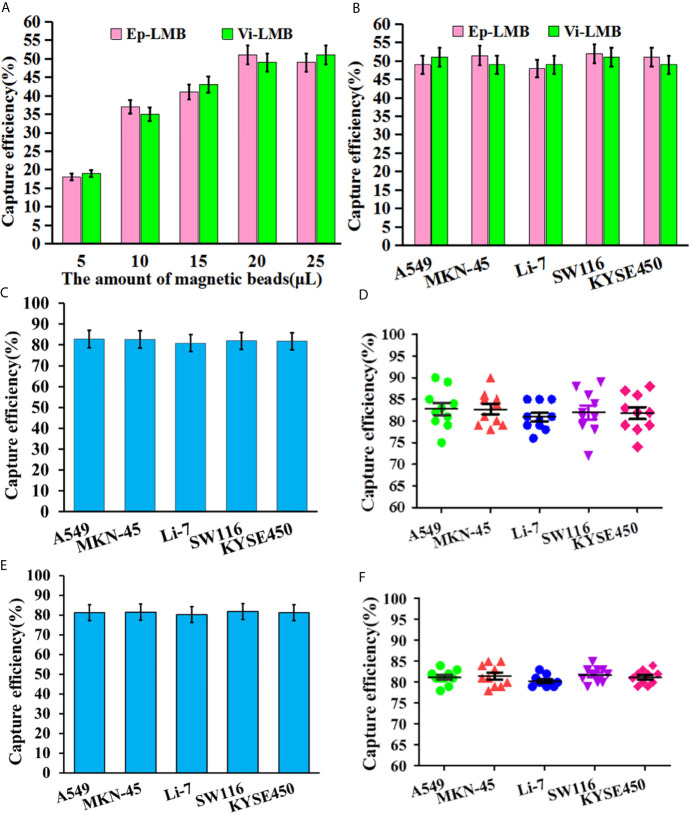
*In vitro* simulation of CTCs capture efficiency: **(A)** The capture efficiency of Ep-LMB and Vi-LMB for A549 cells; **(B)** Capture efficiency of Ep-LMB and Vi-LMB at 20mL for different cancer cell lines; **(C)** Total capture efficiency of Ep-LMB combined with Vi-LMB for different cell lines in PBS; **(D)** Stability of the total capture rate for each cell line in PBS by the combination of Ep-LMB and Vi-LMB; **(E)** Total capture efficiency of Ep-LMB combined with Vi-LMB for different cell lines in blood; **(F)** Stability of the total capture rate for each cell line in blood by the combination of Ep-LMB and Vi-LMB.

The results of *in vitro* simulation of CTCs capture efficiency are shown in **Figure 4**. **Figure 4A** shows the capture efficiency of Ep-LMB and Vi-LMB at different usages for A549 cells in PBS system. The capture efficiency peaked when the usage of Ep-LMB and Vi-LMB was 20μL (51% and 49%, respectively), with no obvious change in capture rate with increasing usage. On the basis of obtaining the optimal usage amount of Ep-LMB and Vi-LMB from A549 cells, the next step was to explore whether there was effect in other cell lines. As shown in **Figure 4B**, there was no obvious difference in the capture efficiency of Ep-LMB and Vi-LMB for MKN-45, Li-7, SW116 and KYSE450 cell lines when compared to that for A549 cells. In order to improve the efficiency of cell capture, Ep-LMB combined with Vi-LMB were used to capture cell lines (**Figure 4C**). A combined usage of Ep-LMB and Vi-LMB resulted in significant increase of the capture rate, and the capture rate for each cell line was over 80%, showing no statistical difference (P>0.05). According to the results of the stability of the total capture rate for each cell line by the combination of Ep-LMB and Vi-LMB (**Figure 4D**), the combined usage resulted in a relatively stable capture efficiency of each cell (average capture rate of >80%). In order to verify the stability in the blood, we perform verification in the blood simulation system, According to the results of the stability of the total capture rate for each cell line by the combination of Ep-LMB and Vi-LMB (**Figures 4E, F**), the combined usage resulted in a relatively stable capture efficiency of each cell (average capture rate of >80%), suggesting the feasibility and stability of this method for CTCs capture in clinical blood samples.

### Capture and Identification of CTCs in Clinical Blood Samples

**Figure 5:**
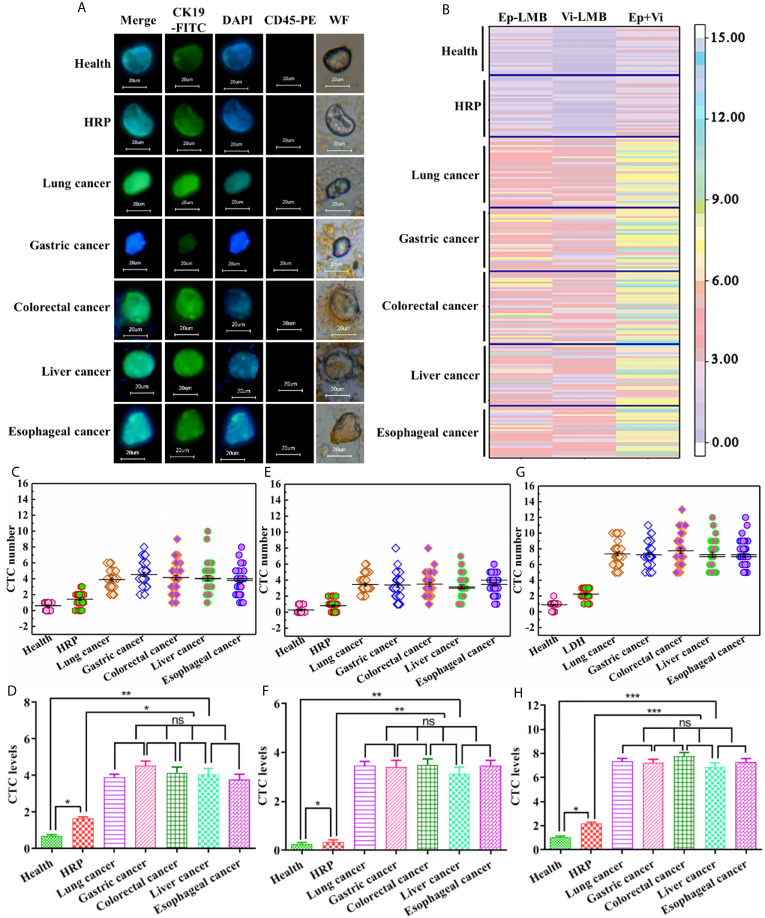
Capture and identification of CTCs in clinical blood samples: **(A)** Immunofluorescence analysis of CTCs in blood samples of healthy subjects, HRP patients and patients with different types of cancer (scale: 20μm); **(B)** Hotspot map of CTCs count captured by Ep-LMB and Vi-LMB in blood samples of healthy subjects, HRP patients and patients with different types of cancer; **(C)** Distribution of Ep-LMB CTCs in blood samples of healthy subjects, HRP patients and patients with different types of cancer; **(D)** Comparison of average count of Ep-LMB CTCs in blood samples of healthy subjects, HRP patients and patients with different types of cancer; **(E)** Distribution of Vi-LMB CTCs in blood samples of healthy subjects, HRP patients and patients with different types of cancer; **(F)** Comparison of average count of Vi-LMB CTCs in blood samples of healthy subjects, HRP patients and patients with different types of cancer; **(G)** Distribution of total CTCs in blood samples of healthy subjects, HRP patients and patients with different types of cancer; **(H)** Comparison of average count of CTCs in blood samples of healthy subjects, HRP patients and patients with different types of cancer. P>0.05 (ns), meant that the difference there was no statistically significant, P < 0.05 (*P < 0.05; **P < 0.01; ***P < 0.001) meant that the difference was statistically significant.

Besides CTCs, cells after magnetic enrichment included a certain number of erythrocyte and leukocyte. At present, the recognition of CTCs was mainly based on the specific expression of CTCs antigen. Thus, CTCs were identified in this experiment by immunofluorescence staining. Under the white light microscope, in the presence of visible cell morphology, strong positive CK19-FITC green fluorescence and DAPI blue fluorescence, and negative CD45 staining, it could be judged that the cell captured by the magnetic bead from the blood was CTCs. **Figure 5A** shows the results of immunofluorescence assay in blood samples of healthy subjects, as well as patients with HRP and different types of cancer. As shown in this figure, CTCs could be captured in blood samples from healthy subjects, as well as patients with HRP and different types of cancer. **Figure 5B** is a hotspot map of CTCs count captured by Ep-LMB and Vi-LMB in blood samples of healthy subjects, as well as patients with HRP and different types of cancer. It was observed that the count of CTCs captured by Vi-LMB and Ep-LMB in the blood samples of cancer patients was significantly higher than that of healthy subjects and patients with HRP. **Figure 5C** shows the distribution of Ep-LMB CTCs in blood samples of healthy subjects, HRP patients and patients with different types of cancer. The Ep-LMB CTCs count in healthy subjects and HRP patients was lower than that in patients with various cancers. Meanwhile, based on **Figure 5D**, there was no significant difference in the average Ep-LMB CTCs count among patients with various cancers (P>0.05). The count of Ep-LMB CTCs in patients with various cancers was statistically different from that in healthy subjects and patients with HRP (P<0.05). Besides, the count of Ep-LMB CTCs in patients with HRP was significantly higher than that in healthy subjects. The average Ep-LMB CTCs count in each group was described as follows: (Av=0.6, N=25) in healthy subjects, (Av=1.4, N=32) in HRP patients, (Av=3.9, N=37) in lung cancer patients, (Av=4.1, N=33) in gastric cancer patients, (Av=4.2, N=38) in colorectal cancer patients, (Av=4.0, N=32) in liver cancer patients, (Av=3.8, N=34) in esophageal cancer patients, and (Av=4.0, N=174) total cancer patients. **Figure 5E** shows the distribution of Vi-LMB CTCs in blood samples of healthy subjects, HRP patients and patients with different types of cancer. The Vi-LMB CTCs count in healthy subjects and HRP patients was lower than that in patients with various cancers. Meanwhile, based on **Figure 5F**, there was no significant difference in the average Vi-LMB CTCs count among patients with various cancers (P>0.05). The count of Vi-LMB CTCs in patients with various cancers was statistically different from that in healthy subjects and patients with HRP (P<0.01). Besides, the count of Vi-LMB CTCs in patients with HRP was significantly higher than that in healthy subjects. The average Vi-LMB CTCs count in each group was described as follows: (Av=0.3, N=25) in healthy subjects, (Av=0.8, N=32) in HRP patients, (Av=3.5, N=37) in lung cancer patients, (Av=3.1, N=33) in gastric cancer patients, (Av=3.6, N=38) in colorectal cancer patients, (Av=2.9, N=32) in liver cancer patients, (Av=3.5, N=34) in esophageal cancer patients, and (Av=3.3, N=174) total cancer patients. **Figure 5G** shows the distribution of total CTCs count in blood samples of healthy subjects, HRP patients and patients with different types of cancer. The total CTCs count in healthy subjects and HRP patients was lower than that in patients with various cancers, CTCs in healthy people are mainly distributed in 0-2, CTCs in HRP patients are mainly distributed in 1-3, and CTCs in cancer patients are mainly distributed in more than 5. Meanwhile, based on **Figure 5H**, there was no significant difference in the average CTCs count among patients with various cancers (P>0.05). The count of CTCs in patients with various cancers was statistically different from that in healthy subjects and patients with HRP (P<0.001). Besides, the count of CTCs in patients with HRP was significantly higher than that in healthy subjects. The average CTCs count in each group was described as follows: (Av=0.9, N=25) in healthy subjects, (Av=2.4, N=32) in HRP patients, (Av=7.4, N=37) in lung cancer patients, (Av=7.2, N=33) in gastric cancer patients, (Av=7.8, N=38) in colorectal cancer patients, (Av=6.9, N=32) in liver cancer patients, (Av=7.3, N=34) in esophageal cancer patients, and (Av=7.3, N=174) total cancer patients.

### Correlation Analysis Between Clinicopathological Parameters of Patients and CTCs Counts

Group *t*-test or one-way analysis of variance were used to analyze the correlation between the CTCs counts in blood samples of the enrolled 174 patients and the clinicopathological parameters of cancer patients, as shown in **Table 1**.

**Table 1:** Correlation between CTCs count and clinicopathological parameters of patients.

Clinicopathological parameters	n	CTCs (x+s)	P
Age (years)			0.503
≤60	55	6.2 ± 0.33	
>60	119	5.9 ± 0.21	
Gender			0.153
Male	102	6.3 ± 0.23	
Female	72	5.7 ± 0.29	
Smoking history			0.718
With	77	5.9 ± 0.27	
Without	97	6.1 ± 0.24	
Distant metastasis			0.032
With	48	8.9 ± 0.30	
Without	126	6.7 ± 0.10	
Tumor stage			<0.041
I+II	118	6.7 ± 0.11	
III+IV	56	8.7 ± 0.28	

**Figure 6:**
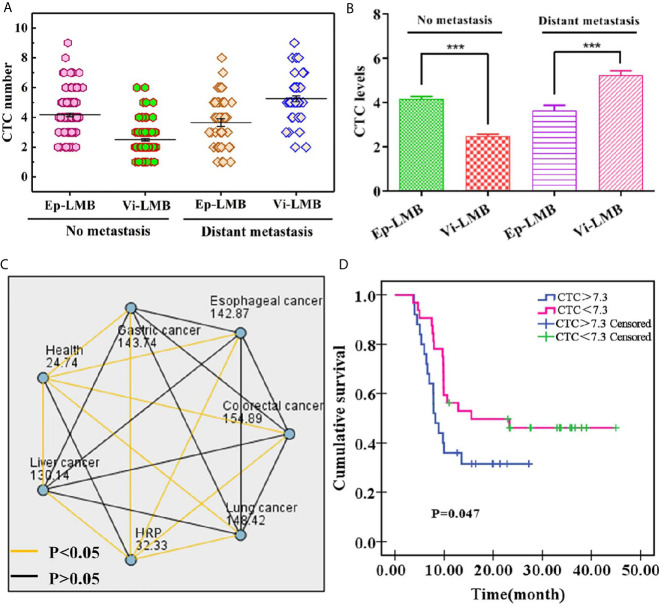
Clinical data of cancer patients: **(A)** Distribution of Ep-LMB CTCs and Vi-LMB CTCs in blood samples of no metastasis and distant metastasis patients; **(B)** Comparison of average count of Ep-LMB CTCs and Vi-LMB CTCs in blood samples of no metastasis and distant metastasis patients; **(C)** Kruskal-Wallis test results between healthy subjects, HRP patients, and cancer patients; **(D)** Survival curve of HRP patients with cancer and those with cancer alone. ***P < 0.001.

According to the analysis, it was found that there was no evident difference in the average count of total CTCs in patients with different age, gender and smoking history (P>0.05), and there was significant difference in the average count of CTCs between patients with and without metastasis (P<0.05). Among them, EpCAM-LMB CTCs in patients without metastasis were significantly higher, and Vi-LMB CTCs in patients with distant metastases were significantly higher, and the differences were statistically significant (P<0.001) (**Figures 6A, B**). The average count of total CTCs was also correlated with tumor stage, which was higher in patients with stage III+IV than those with stage I+II (P<0.05). The count of CTCs in healthy subjects and patients with HRP was significantly lower than that in cancer patients, and there was no significant difference among different cancer patients (**Figure 6C**).

Furthermore, of the enrolled 174 cancer patients, 86 cancer patients were followed up by telephone. Kaplan-Meier survival analysis was performed on the patients, and the results are shown in **Figure 6D**. The survival rate showed a decreased trend with the increase of time, which was significantly lower in CTCs>7.3 than that of CTCs<7.3. The mean survival time of CTCs>7.3 and CTCs<7.3 was 8.21 months and 15.54 months, respectively, which was obviously lower in the former patients when compared with that of the latter patients (P=0.047), this indicates that the survival rate of cancer patients is related to the number of CTCs.

## Discussion

Studies have shown that CTCs can serve as a new diagnostic tool, providing information for tumor staging, prognosis (fewer CTCs count indicates longer survival) and evaluation of efficacy in an earlier stage. As a non-invasive method, CTCs count can also provide reference for the biological characteristics of primary and metastatic tumors. CTCs has been found in prostate cancer, colorectal cancer, breast cancer, lung cancer, ovarian cancer, liver cancer, bladder cancer and other solid tumors, which is becoming an independent predictor of tumor prognosis and response to treatment (26, 27). In terms of breast cancer, the count of CTCs (taking ≥5 CTCs/7.5 ml peripheral blood as the threshold) has been proved to be an independent prognostic indicator, which can well predict the progression-free survival (PFS) and overall survival (OS) of patients with metastatic breast cancer. For colorectal cancer patients receiving first-line treatment, the change of CTCs count before and after treatment can be regarded as a predictor of PFS and OS (28). Furthermore, for patients with advanced gastric and esophageal adenocarcinoma receiving first-line chemotherapy, Sclafani et al. (29) documented that patients with ≥2 CTCs in peripheral blood had poor response to chemotherapy and shorter survival time. Besides, the detection of CTCs has been proved to have the advantages of timeliness and higher accuracy compared with traditional imaging (30). However, the detection of CTC depends on the sensitivity of enrichment technology. The only approved CTC detection method is the CellSearch system, which uses EpCAM coated magnetic beads to enrich CTC and anti-CK antibodies for recognition, but the positive rate of CTC detected by this method is 71.1% (31) because it produces negative results for CTC with EMT. Therefore, we construct a new CTC separation system, and the research shows that the capture efficiency of this system is 4.4 ± 1.2 times higher than that of the Cellsearch system (32). On this basis, we add a marker (vimentin) that can recognize mesenchymal CTC, which solves the defects of CellSearch system. The aforementioned studies support that CTCs is an independent predictor of tumor patients’ prognosis and response to treatment. It is worth studying whether CTCs is related to the knock-on effect caused by other diseases. Prior studies were carried out generally in the detection of CTCs in cancer patients. Whether there is CTCs in the blood of normal people and other patients is an issue that has not been valued in the past. Only a few reports have reported the presence of CTCs in healthy subjects and patients with other diseases. For example, Yue Yu et al. (31) detected that the median of CTCs in blood of healthy subjects was 5.71 [4.03-7.38], which was 6.74 [4.64-8.27] and 10.82 [18.21-15.84] in patients with benign diseases and non-small cell lung tumors, respectively. In a study carried out by Fuming Qi et al. (32) the next year, CTCs were detected by PCR in serum samples of 64 patients with bladder cancer and 20 healthy volunteers by using folate receptor α as tumor marker. It was found that the median of CTCs was 26.5 Cu/3mL in the serum of bladder cancer patients and 14.0 Cu/3mL in the control group. In addition, Fei Zhou et al. (33) reported in 2015 that the CTCs of NSCLC patients was 12.41 ± 9.02/3mL, significantly higher than that of benign lung disease patients (6.95 ± 5.45/3mL, p<0.001) and healthy donors (5.95 ± 4.57/3mL, p<0.001).

The above reports suggest that CTCs can be detected in blood of healthy subjects and patients with other diseases. In addition, a recent report on RA revealed that the death rate of rheumatoid arthritis patients due to infection and respiratory diseases is decreasing in recent decades, while the incidence of malignant tumors is increasing (34). A recent study shows that both males and females with rheumatoid arthritis have an increased risk of malignancy (35). As evidenced by the above description, it can be found that CTCs exist in the blood of both cancer patients and non-cancer patients. Simultaneously, inflammation may also be a potential factor for tumor development (36, 37). Accordingly, the present study compared the differences of CTCs among healthy subjects, patients with HRP and cancer patients. It was detected that the CTCs count was (Av=0.9) in healthy subjects, (Av=2.3) in HRP patients and (Av=7.3, N=174) total cancer patients. The count of CTCs in healthy subjects was significantly lower than that in HRP patients and cancer patients, yet without the detection of statistical difference among patients with various cancers.

To sum up, the study confirmed that CTCs can achieve early tumor detection and auxiliary diagnosis, and its number is related to the occurrence and development of tumors, and CTCs can be detected in HRP and sub-health population. As a liquid biopsy technique, it has important clinical application value and will play a huge role in the field of early tumor detection and auxiliary diagnosis.

## Data Availability Statement

The original contributions presented in the study are included in the article/supplementary material, further inquiries can be directed to the corresponding authors.

## Ethics Statement

The studies involving human participants were reviewed and approved by The Ethics Committee of Zhabei Central Hospital of Shanghai (ZBLL20180823). The patients/participants provided their written informed consent to participate in this study.

## Author Contributions

CYH drafted and wrote the manuscript, SD and CJH revised and reviewed the work, FP, XDL and HJZ collected and analyzed the data, JZ and PS was in charge of the methodology and experiments, XFL and XYW contributed in the resources of the study, CJH administrated the work. CJH, SD and CYH contributed equally to this work. All authors contributed to the article and approved the submitted version.

## Funding

This work was supported by The Jing’an District General Project of Shanghai (2018MS07).

## Conflict of Interest

JZ, XL, XW and PS were employed by Huzhou Lieyuan Medical Laboratory Company Ltd.

The remaining authors declare that the research was conducted in the absence of any commercial or financial relationships that could be construed as a potential conflict of interest.
